# Supplementary material for: Transcriptome Analysis Reveals Vimentin-Induced Disruption of Cell–Cell Associations Augments Breast Cancer Cell Migration
Source: Cells. 2022 Dec 13;11(24):4035. doi: 10.3390/cells11244035 (PMC9776984; doi:10.3390/cells11244035)
Supplement: Supplementary file 1 [file cells-11-04035-s001.zip › DEGs and enrichment analysis.zip!/DEGs and enrichment analysis/WT_VIMvsVector KEGG/ALL/WT_VIMvsVector_ALL.pathway_map.zip!/WT_VIMvsVector_ALL.pathway_map/src/hsa05418.html]

hsa05418


- hsa:4688

- Down regulated genes

ENSG00000116701(-2.69751290628821)

- hsa:1003

- Down regulated genes

ENSG00000179776(-4.78450423698439)

- hsa:3791

- Down regulated genes

ENSG00000128052(-2.65973347544558)

- hsa:2947

- Down regulated genes

ENSG00000241720(-1.93726005347488)
- hsa:2946

- Down regulated genes

ENSG00000213366(-3.27565378318694)

- hsa:1906

- Down regulated genes

ENSG00000078401(-3.03994258238563)

- hsa:3674

- Up regulated genes

ENSG00000005961(3.91653577454454)

- hsa:596

- Up regulated genes

ENSG00000171791(2.03324203294485)

- hsa:1003

- Down regulated genes

ENSG00000179776(-4.78450423698439)

- hsa:3791

- Down regulated genes

ENSG00000128052(-2.65973347544558)

Close
